# Author Correction: Clear and transparent nanocrystals for infrared-responsive carrier transfer

**DOI:** 10.1038/s41467-019-09888-2

**Published:** 2019-04-17

**Authors:** Masanori Sakamoto, Tokuhisa Kawawaki, Masato Kimura, Taizo Yoshinaga, Junie Jhon M. Vequizo, Hironori Matsunaga, Chandana Sampath Kumara Ranasinghe, Akira Yamakata, Hiroyuki Matsuzaki, Akihiro Furube, Toshiharu Teranishi

**Affiliations:** 1 Institute for Chemical Research, Kyoto University, Gokasho, Uji, Kyoto 611-0011 Japan; 2 Department of Chemistry, Graduate School of Science, Kyoto University, Gokasho, Uji, Kyoto 611-0011 Japan; 3 Graduate School of Pure and Applied Sciences, University of Tsukuba, 1-1-1 Tennodai, Tsukuba, Japan; 4 Graduate School of Engineering, Toyota Technological Institute, 2-12-1 Hisakata, Tempaku, Nagoya 468-8511 Japan; 5 National Institute of Advanced Industrial Science and Technology (AIST), Tsukuba Central 2, 1-1-1 Umezono, Tsukuba, Ibaraki 305-8568 Japan; 6 Department of Optical Science, Tokushima University, 2-1, Minamijosanjima-cho, Tokushima, 770-8506 Japan

**Keywords:** Light harvesting, Electron transfer, Nanophotonics and plasmonics, Nanoparticles

Correction to: *Nature Communications* 10.1038/s41467-018-08226-2, published online 24 January 2019

The original version of this Article omitted the fourth author Taizo Yoshinaga, who is from the ‘Graduate School of Pure and Applied Sciences, University of Tsukuba, 1-1-1 Tennodai, Tsukuba, Japan’. Consequently, the third sentence of the Author Contributions, ‘M.S. and M.K. synthesized the ITO NCs and ITO/semiconductor oxides’ was revised to ‘M.S., M.K. and T.Y. synthesized the ITO NCs and ITO/semiconductor oxides’. This has been corrected in both the PDF and HTML versions of the Article.

