# Supplementary material for: Clear and transparent nanocrystals for infrared-responsive carrier transfer
Source: Nat Commun. 2019 Jan 24;10:406. doi: 10.1038/s41467-018-08226-2 (PMC6345985; doi:10.1038/s41467-018-08226-2)
Supplement: Supplementary file 1 — Supplementary Information [file 41467_2018_8226_MOESM1_ESM.pdf]

Supplementary information

Clear and Transparent Nanocrystals for Infrared-Responsive Carrier Transfer

Sakamoto et al.

Supplementary Notes

Synthetic details

SnO_2 NCs with different sizes: ITO nanocrystals (NCs) were adsorbed onto different-sized SnO_2. We used SnO_2(S) (Wako, nominal diameter is 22–43 nm, which is used for experiments in main text) and SnO_2(L) (US Research Nanomaterials, Inc., nominal diameter is 450 nm). The size distribution and TEM images of different-sized SnO_2 are provided in supplementary Fig. 10. The average diameter of SnO_2(S) and SnO_2(L) estimated by TEM image are 27 ± 13, and 276 ± 155 nm, respectively.

Loading of ITO NCs onto SnO_2 NCs with different sizes: Different-sized SnO_2 (100 mg) and sebacic acid (1 mmol) were added into the chloroform solution (50 mL) containing the desired quantity of ITO NCs (10-wt%-Sn doping), followed by stirring for 24 h. Both ends of carboxyl group in a sebacic acid work as linker of ITO NCs and SnO_2. The solution was filtered and washed with hexane and chloroform. The pasted samples are calcined in air at 600°C for 30 min, and then are annealed under reductive atmosphere (4% H_2/Ar) at 280°C for 5 h to regenerate the LSPR. TEM measurements revealed no changes in size of the ITO NCs upon heat treatments (supplementary Fig. 4). All ITO/oxide show an LSPR peak in the NIR region, even after annealing under reductive atmosphere (Figure 1c).

Estimation of instrument response function (IRF): The FWHM of IRF in the system for transient absorption measurement at the ps scale was estimated from the transient absorption signal of Si wafer measured by the present system.[1] The transient absorption of Si appears upon the irradiation of laser pulse without delay. In addition, the decay of the transient absorption of Si is sufficiently long. Therefore, the transient absorption signal of Si can be assumed as a step function. Based on the above hypothesis, the FWHM of IRF in the system can be estimated from the derivation of the transient absorption signal of Si wafer.

Investigation on the decay profiles of ITO/metal oxide upon the excitation of LSPR in ps region: We measured the decay rates of LSPR signal of ITO/Metal Oxides in ps region (Supplementary Fig. 7 and Table 1). The lifetime of hot electrons in ITO/SiO_2 is longer than those in ITO/other metal oxides. This difference corresponds the low thermal conductivity of SiO_2 in comparison with the other metal oxides (Table 2). Hamanaka, Y. *et al.,* reported that the thermal conductivity is one of the important parameter governing the relaxation dynamics of plasmonic Au NCs. According to their research, the matrix of low thermal conductivity, such as SiO_2, suppresses the

relaxation and extend the lifetime of excited AuNCs.[2] We consider that the similar phenomenon was observed in the present system.

Investigation on the decay profiles of ITO/SnO_2 with different sizes upon the excitation of LSPR in μs region: We investigate the charge separation process between ITO NCs and different-sized SnO_2 NCs (SnO_2(S) and SnO_2(L) (Supplementary Figs. 11-13 and Table 3). The time-resolved IR spectrum of ITO/SnO_2(S) and ITO/SnO_2(L) after the excitation LSPR band of ITO are shown in supplementary Fig. 11. At 5 μs after excitation, the spectrum of overlapping of free carrier absorption (FCA) of SnO_2 and trapped carrier peaks at around 3800 nm was observed. We observed the clear FCA spectrum in ps region (Figure 2b). Therefore, this result indicated that the FCA in SnO_2 generated by the electron injection form ITO NCs decays via charge recombination and trapping. Supplementary Fig. 13 shows the decay profile of transient absorption at 5,000 nm. The decay profiles of ITO/SnO_2(S) and ITO/SnO_2(L) were fitted well with the triple-exponential decay function (Supplementary Table 3). The fastest and slowest components increased with increasing size of SnO_2. The multiple decay kinetics depending on the size of SnO_2 could correspond to the different diffusion process of electron concerning the different sizes of SnO_2 and defects trapping and de-trapping processes on SnO_2.[1,7,8] Since the trapping state in the ITO/SnO_2 system estimated by the absorption edge is 0.25 eV from conduction bands minimum, the electron in the shallow trapped state could be retrieved as external energy.[7]

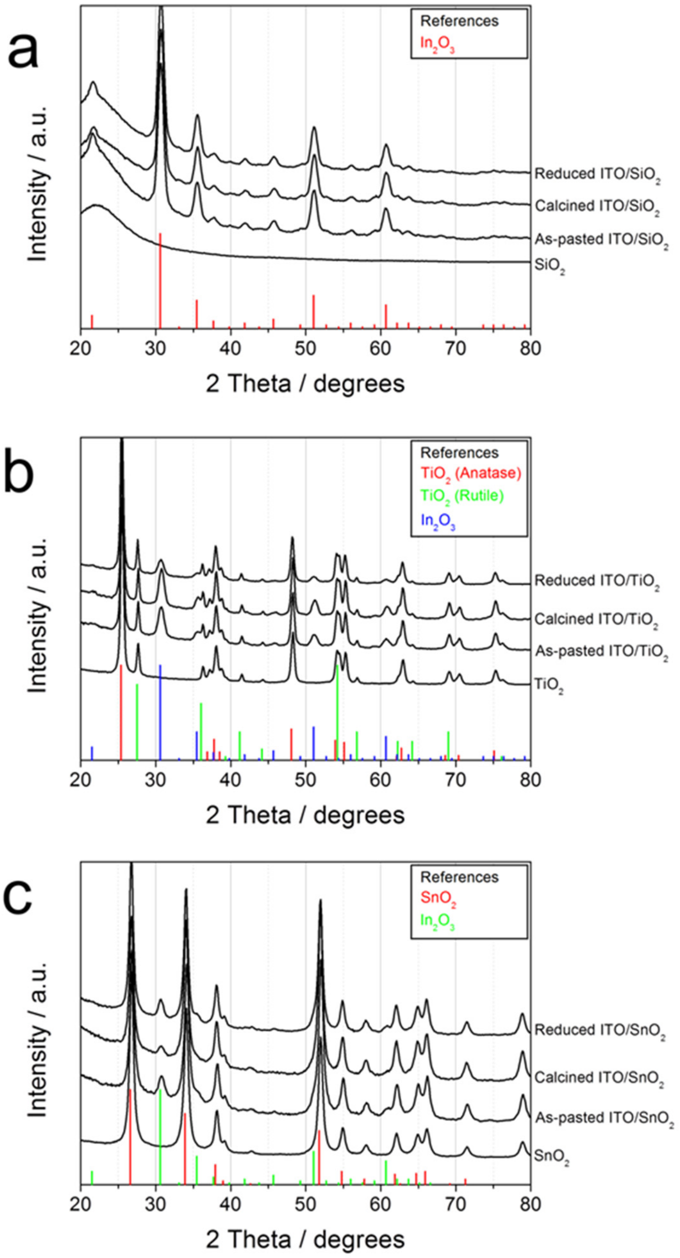

Supplementary Figure 1 | XRD patterns of the ITO/metal-oxide electrode. XRD patterns of **a** ITO/SiO_2, **b** ITO/TiO_2 (P25) and (c) ITO/SiO_2 before and after thermal annealing under air at 600°C for 30 min and reductive annealing under 4% H_2/Ar at 280 °C for 5 h. No shift in diffraction peak was observed during the thermal-annealing and reductive-annealing processes.

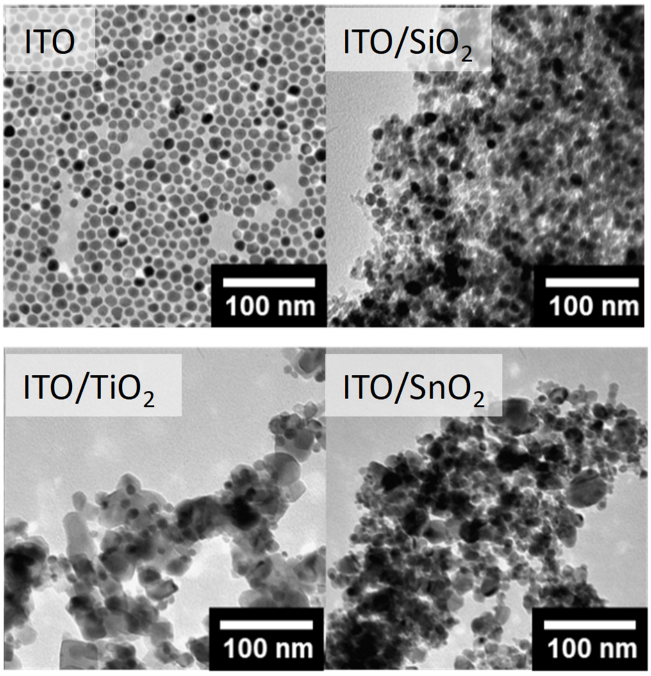

Supplementary Figure 2 | TEM images of ITO-NC/metal-oxide. TEM images of ITO NCs, ITO/SiO_2, ITO/TiO_2 and ITO/SnO_2. No coalescence or aggregation of the ITO NCs were observed before and after thermal and reductive annealing.

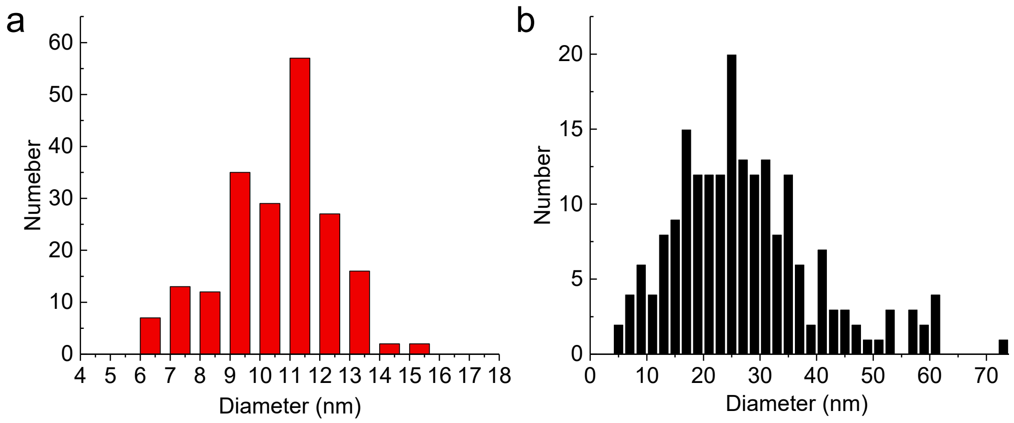

Supplementary Figure 3 | Size distribution of NCs. Size distribution of **a** ITO NCs and **b** SnO_2.

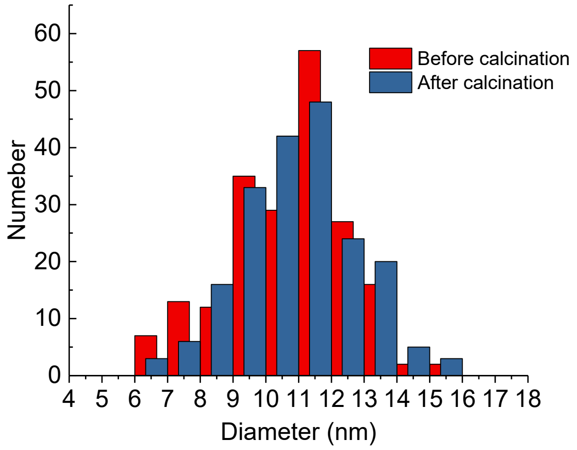

Supplementary Figure 4 | Size distribution of ITO NCs before and after calcination. No change in size of the ITO NCs were observed before and after thermal and reductive annealing.

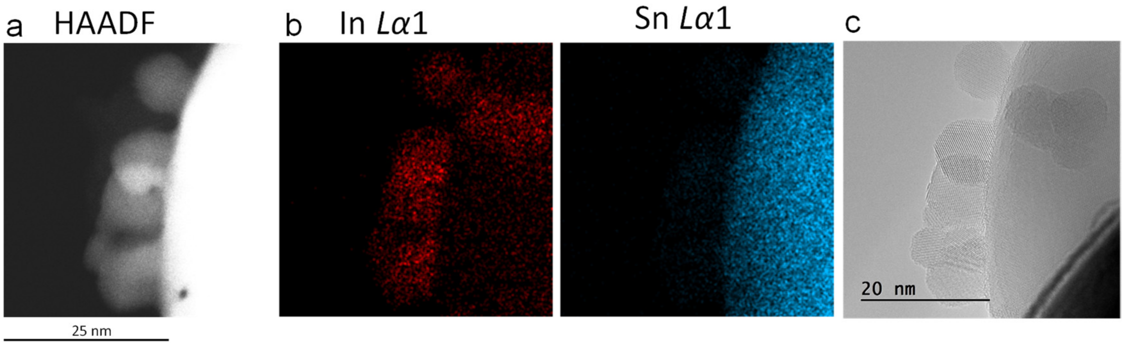

Supplementary Figure 5 | TEM and elemental mapping images of ITO/SnO_2(L) interfaces. a HAADF-STEM image of ITO/SnO_2(L) heterointerface. **b** HAADF-STEM-EDS elemental mapping images of ITO/SnO_2(L) heterointerface. **c** HRTEM image of ITO/SnO_2(L) heterointerface.

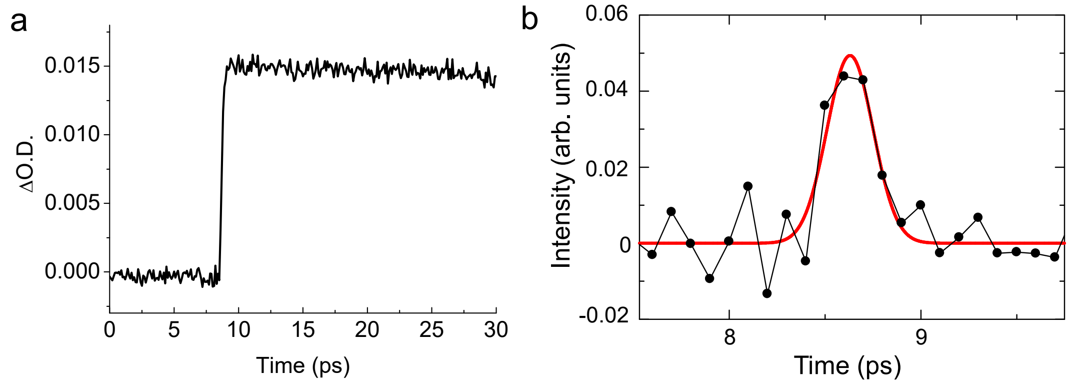

Supplementary Figure 6 | Estimation of IRF in the system for transient absorption measurement at the ps scale.[1] **a** transient absorption signal in Si wafer measured by the present system. **b** the derivation of the transient absorption signal. Red line is a best fit. The FWHM value of IRF was estimated to be 285 ± 40 fs by the fitting of the curve by using the Gauss function (red line).

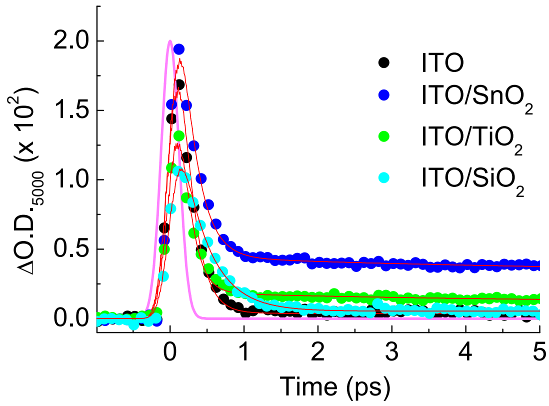

Supplementary Figure 7 | Kinetic profiles of transient absorption of ITO and ITO/metal oxides in ps region. Kinetic profiles of transient absorption of ITO and ITO/metal oxides at the ps scale at 5,000 nm upon the excitation by a 1,700-nm laser Instrument response function (IRF) (FWHM=285 ± 40 fs) are shown by pink line. The kinetic profiles were fitted by double exponential decay function with the constant component: $A_1\exp(-t/\tau_1) + A_2 \exp(-t/ \tau_2)$ +const. ($t \geq 0$) convoluted by IRF. The red lines are best fit. The errors are estimated by uncertainties of FWHM of IRF (285 ± 40 fs) and time origin (± 0.05 ps).

Supplementary Table 1 | Decay (τ_1 and τ_2) components of LSPR in the ps region.

	τ_1 (ps)	τ_2 (ps)
ITO/SiO_2	0.37 ± 0.11	NA
ITO/SnO_2	0.21 ± 0.10	5.3 ± 0.7
ITO/TiO_2	0.14 ± 0.09	4.5 ± 0.7
ITO	0.17 ± 0.08	NA

Supplementary Table 2 | Thermal conductivity of metal oxide and decay component (τ_1) of LSPR in the ps region.

	Thermal conductivity of metal oxides (W/mK)	τ_1 (ps)
ITO/SiO_2	1.3[3]	0.37 ± 0.11
ITO/SnO_2	10[4]	0.21 ± 0.10
ITO/TiO^2	11[5]	0.14 ± 0.09
ITO	-	0.17 ± 0.08

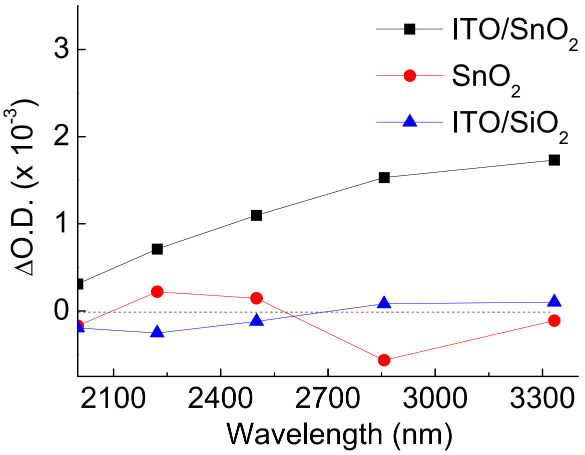

Supplementary Figure 8 | TR-IR spectrum of ITO/SnO_2, ITO/SiO_2, and SnO_2 at 5 μs following excitation by a 1,400-nm laser. No FCA was observed after laser irradiation in the cases of ITO/SiO_2, ITO/TiO_2 and SnO_2.

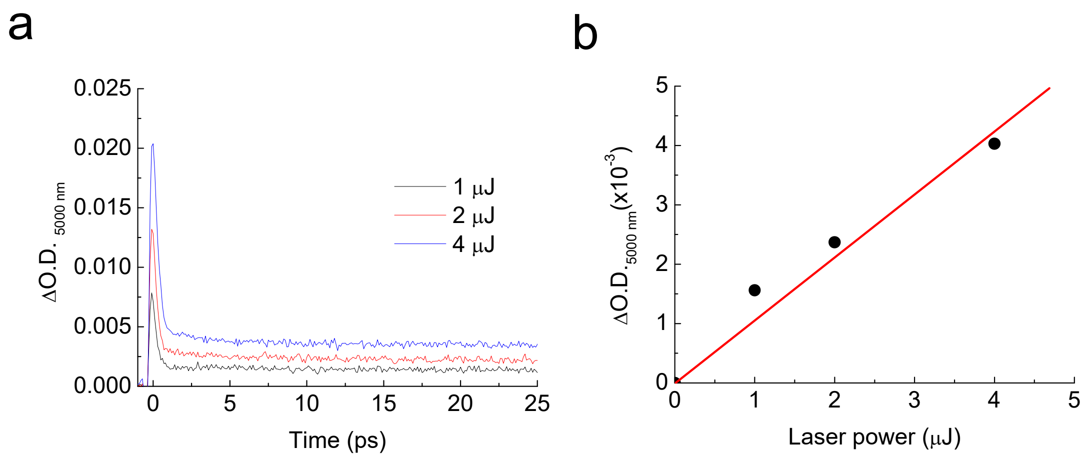

Supplementary Figure 9 | Laser-power dependence of the intensity of the FCA absorption of SnO_2 at 5,000 nm. a The decay profiles of ITO/SnO_2 at 5,000 nm upon excitation of a 1,700-nm laser with different powers. **b** Plots of laser-power dependence of ΔOD at 5,000 nm of ITO/SnO_2 film at 10 ps after excitation. The slope of the plot is 1, indicating that carrier injection is a one-photon process.

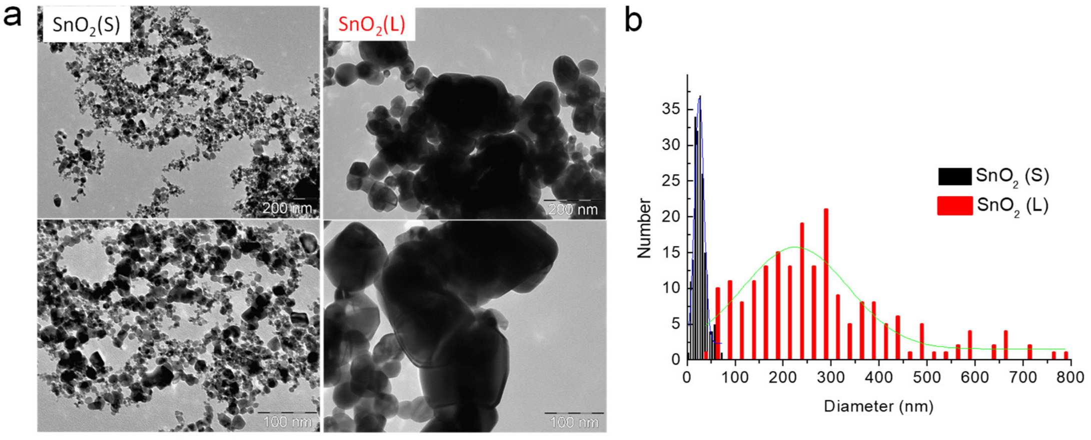

Supplementary Figure 10 | The SnO_2 with different sizes. a The TEM images of different sized SnO_2 nanocrystal (SnO_2(S) and SnO_2(L)). **b** Size distribution of SnO_2(S) and SnO_2(L).

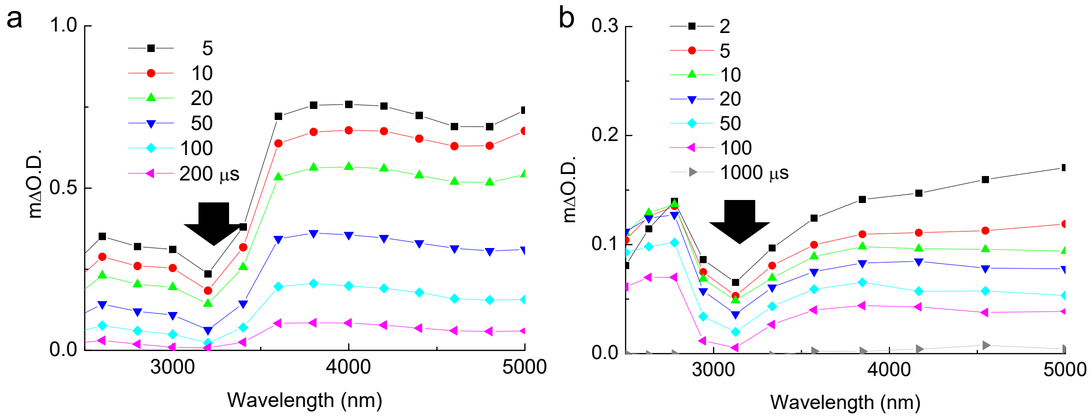

Supplementary Figure 11 | Time-resolved IR spectrum of ITO/SnO_2 with different sizes. Time-resolved IR spectrum of **a** ITO/SnO_2(S) and **b** ITO/SnO_2(L). The dipping feature from 2600 to 3600 nm, which is indicated by black arrow, is affection of absorption of water.[6]

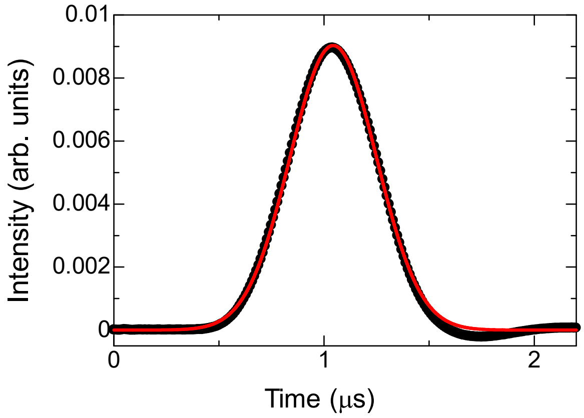

Supplementary Figure 12 | Estimation of instrument response function of μs-transient absorption measurement system. The scattering of laser pulse measured by the present system. Red line is a best fit. The FWHM value of IRF was estimated to be 0.485 μs by the fitting of the curve by using the Gauss function (red line).

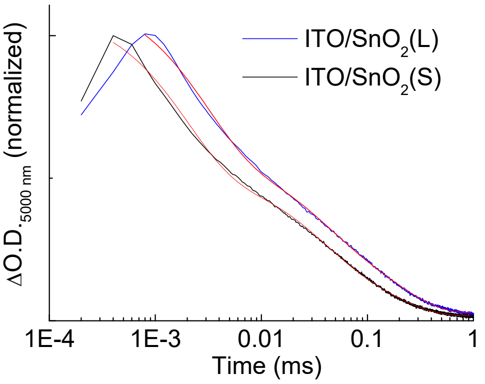

Supplementary Figure 13 | The kinetic profile of ITO/SnO_2 in the μs region. The kinetic profiles of ITO/SnO_2(S) and ITO/SnO_2(L) at 5,000 nm in the μs region after an excitation by the 1,400-nm laser (the FHWM of IRF: 0.485 μs). The red line shows the best fit.

Supplementary Table 3 | Decay components of transient absorption at 5000 nm of ITO/SnO_2 with different sizes.

	τ_1 (μs)	τ_2 (μs)	τ_3 (μs)
ITO/SnO_2(S)	2.0 ± 0.1	33 ± 1	160 ± 1
ITO/SnO_2(L)	3.2 ± 0.4*	31 ± 2.8	179 ± 4.2

* The value affected by the light scattering caused by the large SnO_2 particles.

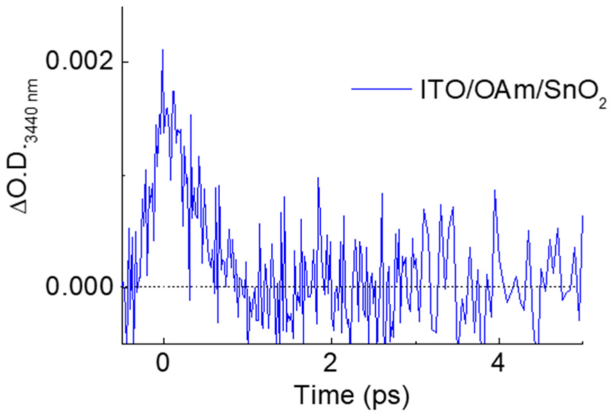

Supplementary Figure 14 | Kinetic profile of the transient absorption of ITO/OAm/SnO_2 at a 3,440-nm upon excitation by the 1,700-nm laser. Only a signal related to ultrafast-LSPR-induced decay was observed. Formation of FCA was not observed, indicating that electron injection was inhibited by the insulating layer.

Supplementary references

1. Du, L. Furube, A. Yamamoto, K. Hara, K. Katoh, R. Tachiya, M. Plasmon-Induced Charge Separation and Recombination Dynamics in Gold-TiO_2 Nanoparticle Systems: Dependence on TiO_2 Particle Size, *J. Phys. Chem. C* **113**, 6454, (2009).

2. Hamanaka, Y. Kuwabata, J. Tanahashi, I. Omi, S. & Nakamura, A. Ultrafast electron relaxation via breathing vibration of gold nanocrystals embedded in a dielectric medium, *Phys. Rev. B* **63**, 104302, (2001).

3. Andersson, S. & Dzhavadov, L. Thermal conductivity and heat capacity of amorphous SiO_2: pressure and volume dependence, *Journal of Physics: Condensed Matter,* **29**, 6209-6216 (1992).

4. Li, S. *et al.,* Thermal conductivity of individual tin dioxide nanobelt, *Appl. Phys. Lett.* **84**, 2638, (2004).

5. Touloukian, Y. S. Powell, R. W. Ho, C. Y. & Klemens, P. G. *Thermophysical Properties of Matter - The TPRC Data Series. Volume 2. Thermal Conductivity - Nonmetallic Solids,* 1971.

6. Hale, G. H. & Querry, M. R. Optical Constants of Water in the 200-nm to 200-μm Wavelength Region, *Applied Optics*, **12**, 555-563, (1973).

7. Du, L., Furube, A., Hara, K., Katoh, R. & Tachiya, M. Ultrafast plasmon induced electron injection mechanism in gold–TiO_2 nanoparticle system. *J. Photochem. Photobiol. C.* **15**, 21-30, (2013).

8. Yamakata, A., Vequizo, J. J. M., Matsunaga, H. Distinctive Behavior of Photogenerated Electrons and Holes in Anatase and Rutile TiO_2 Powders, *J. Phys. Chem. C* **119,** 24538 –24545, (2015).
